# Scalable screening for structural heart disease: promises from artificial intelligence-electrocardiogram tools

**DOI:** 10.1093/ehjdh/ztaf048

**Published:** 2025-05-27

**Authors:** Charalambos Antoniades, Kenneth Chan

**Affiliations:** Acute Multidisciplinary Imaging & Interventional Centre, British Heart Foundation (BHF) Centre of Research Excellence, Division of Cardiovascular Medicine, Radcliffe Department of Medicine, NIHR Oxford Biomedical Research Centre, University of Oxford, Oxford, OX3 9DU, UK; Acute Multidisciplinary Imaging & Interventional Centre, British Heart Foundation (BHF) Centre of Research Excellence, Division of Cardiovascular Medicine, Radcliffe Department of Medicine, NIHR Oxford Biomedical Research Centre, University of Oxford, Oxford, OX3 9DU, UK

**This editorial refers to ‘Development and Multinational Validation of an Ensemble Deep Learning Algorithm for Detecting and Predicting Structural Heart Disease Using Noisy Single-lead Electrocardiograms’, by A. Aminorroaya *et al.*, https://doi:10.1093/ehjdh/ztaf034.**

## Introduction

The global burden of structural heart diseases (SHDs) is escalating over the last decade due to the aging population, where chronic exposure to cardiovascular risk factors (such as hypertension) is partly accountable for driving this increase.^1^ Early detection of SHD could provide a window of opportunity for timely prevention interventions.^2^ Indeed, ∼1 in 20 adults aged over 45 years have asymptomatic left ventricular systolic dysfunction (LVSD),^3^ 1 in 10 adults aged 65 or older have moderate/severe valvular heart disease (VHD),^4^ and 1 in 5 hypertensive individuals have left ventricular hypertrophy (LVH), conditions that are largely preventable.^5^ There is a need for a scalable and easily accessible tool to facilitate more targeted screening, guide downstream investigations and enable the timely deployment of therapeutic measures to modify the natural history of the disease.

The advent of artificial intelligence (AI)-assisted analysis of medical data is revolutionizing all areas of cardiovascular medicine,^6^ from risk prediction^7,8^ to the interpretation of medical images^7^ or electrocardiograms (ECGs).^9^ There is a plethora of AI-ECG tools capable of detecting a wide range of SHDs, such as LVSD, hypertrophic cardiomyopathy, amyloidosis and mitral valve prolapse.^10^ Nevertheless, most AI-ECG tools are based on 12-lead ECG, which may not always be readily available in community settings. This limitation provides the rationale for adapting artificial intelligence models to noisy single-lead or wearable electrocardiogram devices, thereby potentially broadening the scope of SHD screening to larger populations. Artificial intelligence tools utilizing single-lead ECG have previously shown to be useful in detecting severe LVSD and predicting the onset of heart failure before the manifestation of clinical symptoms.^11^

In this issue of the *EHJ Digital Health*, Aminorroaya *et al.*^12^ developed an ensemble of AI-ECG algorithms to detect a broad range of SHDs. Using over 99 000 paired transthoracic echocardiograms and images of single-lead ECG, convolutional neural networks (CNNs) were trained to detect the presence of six SHD conditions, respectively, including LVSD, moderate or severe left-sided valvular disease, moderate or severe aortic regurgitation, moderate or severe aortic stenosis, moderate or severe mitral regurgitation, and severe LVH. The outputs of these CNNs were then integrated with the patient’s age and sex into a deep-learning model, named ADAPT-HEART, to predict cross-sectional SHD. Notably, the ADAPT-HEART model demonstrated good performance in external validation cohorts, comprising a clinically and demographically diverse population drawn from four community hospitals in the USA (a total of ∼44 000 patients) and a population Cohort in Brazil (ELSA-Brazil). The model achieved an excellent area under curve of >0.85 in predicting cross-sectional SHDs.^12^ Furthermore, the model’s value in predicting incident SHD was evaluated in an additional UK Biobank cohort. This further demonstrates the model’s robustness and generalizability in ethnically diverse populations, which is an important aspect considering the widely recognized racial variations in ECG patterns.

## Discussion

**Figure 1:**
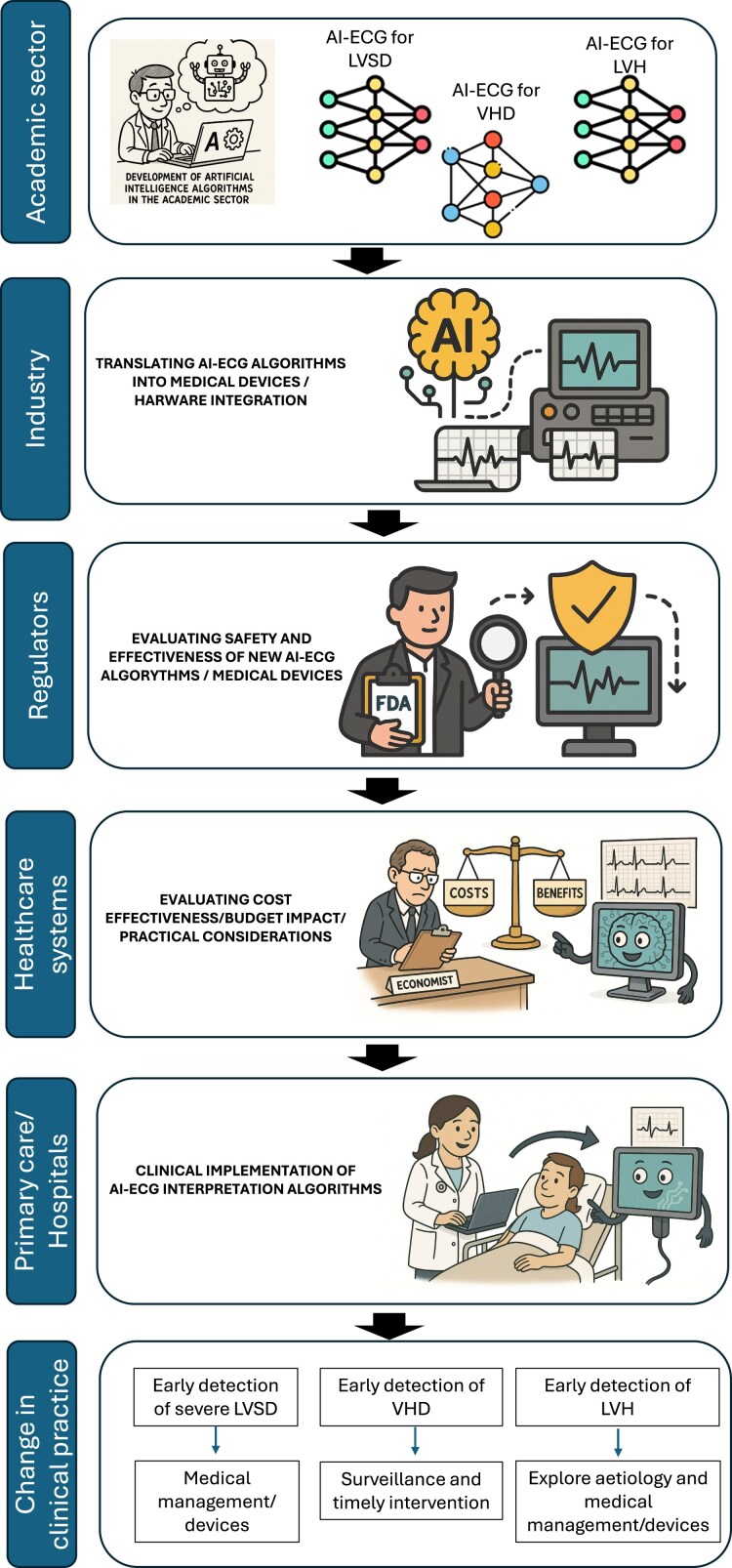
Translating artificial intelligence-electrocardiogram models from research tools to medical devices that impact clinical care. AI, artificial intelligence; ECG, electrocardiogram; LVH, left ventricular hypertrophy; LVSD, left ventricular systolic dysfunction; VHD, valvular heart disease.

The present study enables the possibility of opportunistic screening using a single-lead ECG for detecting a range of SHDs. This has the potential to enhance accessibility, particularly in community settings where clinical ECG measurements may not be readily available. However, questions remain about the utility of this AI-ECG model across different hardware platforms. The quality of the ‘single-lead’ ECG extracted from lead I of the 12-lead ECGs in this study may vary significantly from the noisy ECG waveform obtained from peripheral devices, which are often affected by muscle artefacts and impedance from dry skin contact.^13^ Although the authors have introduced artificial noise to adapt some of the expected artefacts, it is unclear whether it truly reflects the different types and magnitudes of noise in real-world ECG peripheral devices.^13^ Furthermore, denoising algorithms are often applied to raw ECG waveforms from mobile devices to varying intensities, which profoundly alter the signal-to-noise ratio and interpretability.^14^ This underscores the complexity of vendor-specific hardware platforms, and the need for comprehensive prospective validation of the current AI-ECG tools using real-world single-lead ECG data from a variety of wearable devices before large-scale deployment for SHD screening in the community.^15^ The involvement of industry at this stage is essential, as it can serve as a vehicle for transforming these algorithms into usable applications integrated into hardware. The safety and effectiveness of these applications will need to be evaluated by regulators and approved as medical devices suitable for clinical use (*Figure 1*).

Another key question is about the downstream implications of the screening results. The World Health Organization’s principles of screening state that there should be an accepted treatment for the condition. While the detection of LVSD may trigger established diagnostic and therapeutic pathways, viable treatment options may be lacking. Similarly, the management of otherwise asymptomatic patients with moderate VHD or left ventricular hypertrophy remains poorly defined.^2^ Current evidence regarding the prognostic advantages of treating asymptomatic VHD patients remains debateable,^2^ questioning the value of discovering a substantial proportion of patients with milder phenotypes of SHD. The present study included all patients, regardless of symptom status, thereby raising pertinent questions about whether the results of screening should be stratified based on patients’ age group and clinical presentation to enhance their clinical applicability.

Beyond clinical considerations, it is essential to assess the capacity of healthcare systems not only to deploy this kind of screening but also to cope with the downstream investigations triggered by it. The effectiveness of a screening programme is significantly influenced by the healthcare infrastructure and economic resources available within a specific system (*Figure 1*). In publicly funded systems, widespread AI-ECG screening has the potential to increase the burden of diagnostic and surveillance tests substantially. In contrast, financial constraints may restrict equitable access to follow-up care in privatized systems. Further cost-effectiveness analysis against clinical benefits is essential to evaluate the impact of implementing screening with such AI-ECG tools (*Figure 1*).

In conclusion, this study demonstrated the use of single-lead ECG as a scalable solution to justify opportunistic screening in the era of wearable ECG technologies. Nevertheless, several technical challenges persist, particularly regarding generalisability across various hardware platforms in practical applications. Moreover, the implications of screening results require thorough evaluation within the framework of the healthcare system. Ultimately, further research is essential to establish the health economic advantages, thereby ensuring that AI-ECG screening is both clinically efficacious and financially sustainable.
